# Relationship between soccer physical activity enjoyment and internet addiction among adolescents: the chain mediating effect of emotional self-efficacy and athlete participation

**DOI:** 10.3389/fpsyg.2025.1557282

**Published:** 2025-07-15

**Authors:** Liang Shan, Xiang Che, Haopeng Wang, Anzu Li, Ning Ma, Yizhou Shui

**Affiliations:** ^1^School of Physical Education, Shaanxi Normal University, Xi'an, China; ^2^School of Psychology, Shaanxi Normal University, Xi’an, China; ^3^College of Physical Education, Northwest Normal University, Lanzhou, Gan Su, China

**Keywords:** soccer, adolescents, physical activity enjoyment, internet addiction, chain mediation model

## Abstract

To explore the mechanism of adolescent school soccer players’ participation in Internet addiction behavior, this study surveyed 579 young soccer players from 18 primary and secondary schools in Shaanxi Province using the soccer athlete participation scale, physical activity enjoyment scale, emotional self-efficacy scale, and Internet addiction scale. The participants included 310 males, 260 females, 411 primary school students (8–12 years), and 159 junior high school students (12–15 years). The results show that: (1) Internet addiction is negatively correlated with the enjoyment of participating in soccer. (2) Through the mediation of soccer participation, soccer’s physical active enjoyment predicts Internet addiction. (3) Soccer physical actively enjoyment negatively predicts Internet addiction through the mediation of emotional self-efficacy. (4) Negative prediction of Internet addiction through the chain mediation of soccer athlete participation and emotional self-efficacy. This study constructed a chain mediating model with athlete participation and emotional self-efficacy as key mediating variables. This study provides a theoretical and practical basis for the intervention of Internet addiction through physical activity enjoyment and soccer athlete participation.

## 1 Introduction

Internet addiction (IA) describes prolonged, excessive Internet use that results in reliance and a lack of concern for life’s other necessities. The IA includes reliance, difficulty quitting, heightened tolerance to the Internet, and intense impulses to engage in online activities (Griffiths, 1998). With the accelerated advancement of urbanization, internet services have become increasingly ubiquitous while their costs continue to decline. Concurrently, digital platforms’ content ecosystems are saturated with highly addictive information products (Ko et al., 2022). Meanwhile, as global internet penetration rates steadily rise, internet addiction has evolved into a significant public health challenge transcending age, occupation, and social strata (Hassan et al., 2020). Internet addiction is associated with depression (Lau et al., 2018), anxiety (Cai et al., 2021), insomnia (Goel et al., 2021) and other psychological symptoms and mental disorders (Otsuka et al., 2020). It is often accompanied by academic difficulties (Kuo et al., 2018), social withdrawal (Kato et al., 2020) and aggressive behavior (Zhao et al., 2022). These symptoms can impair the academic performance of adolescents, reduce their quality of life, and lead to serious social adaptation difficulties. The IA issues also have a very serious influence on adolescent health development (Jiang et al., 2024). In the Internet age, the phenomenon of adolescents’ IA has become increasingly prominent. When some adolescents face the pressure of study and life or the dilemma of being “unsatisfactory,” they are easy to turn their spiritual support to the network, and then evolve into excessive dependence. By December 2021, the number of Internet users aged between 10 and 19 in China has reached 137 million, accounting for 13.3 percent of all Internet users (Pagliara et al., 2023).

Faced with the increasingly serious problem of adolescent Internet addiction, scholars in many fields have conducted in-depth research from multiple levels and angles and reached a consensus that the root cause of Internet addiction is mainly rooted in the psychological level. Specifically, factors such as negative personality traits (Lei et al., 2006; Yang et al., 2006), negative coping strategies (Yildirim Demirdöğen et al., 2024), depressive mood tendency (Young and Rodgers, 1997), high social anxiety (Wang et al., 2025), lack of self-esteem (Liang et al., 2024), loneliness, and learning burnout (Li S. et al., 2023; Ge et al., 2025), together constitute the leading cause for the Internet addiction dilemma. Therefore, in this study, we hope to alleviate the phenomenon of adolescent internet addiction by using soccer as an entry point.

As a team sport, soccer is loved by adolescents all over the world. It is not only a fundamental physical activity, but also a way of education with profound influence (Qader et al., 2017; Pifer et al., 2018; Park et al., 2020). For adolescents, their physical and mental aspects in the development period, sports are an essential way to promote the healthy growth of them. Studies have also shown that sports can enhance adolescents’ self-control ability and emotional self-efficacy, and soccer can help improve adolescents’ self-control ability (Andretta and McKay, 2020).

Chinese adolescents are usually faced with great academic pressure, and lack of time for sports can only rely on the Internet to relieve their pressure, thus forming IA. Most people with IA have both psychological and physiological negative emotions (Pontes, 2017; Guo et al., 2019; Shoukat, 2019). Adolescents use sports to achieve both physical and psychological fulfillment.

### 1.1 Physical activity enjoyment and IA

Physical activity enjoyment is closely linked to the goals of the positive psychology movement as a positive emotional experience that not only promotes the overall development of adolescents but also significantly improves their quality of life (Baceviciene et al., 2019). What’s more, it helps to reduce negative emotions, such as IA. Adolescents who like physical exercise more are likely to be happier, and have more self-efficacy and confidence, which helps them deal with negative emotions like sadness and anxiety and lead a more active and healthy lifestyle (Garn et al., 2016). Physical activity enjoyment can help to improve the mental health of individuals, thus reducing the possibility of IA. These positive changes in mental health can make adolescents more motivated to face challenges and difficulties in real life, rather than relying on the Internet. Based on the combing analysis of previous studies, this study found that exercise pleasure may have an impact on Internet addiction. Therefore, hypothesis 1 is proposed.

*H1*: Adolescent soccer physical activity enjoyment directly affects adolescents' IA.

### 1.2 Mediating role of emotional self-efficacy

Self-efficacy refers to an individual’s confidence in their ability to complete various tasks and challenges. This positive mindset, which involves believing in one’s capability to handle different situations and achieve success, is known as the theory of self-efficacy (Bandura and Wessels, 1994). Emotional self-efficacy, on the other hand, pertains to an individual’s ability and confidence in managing and controlling their emotions. Due to the incomplete psychological development of adolescents, their emotional regulation skills are significantly limited. When faced with negative emotions, they often resort to non-adaptive coping strategies such as avoidance or indulgence, which can further undermine their emotional self-efficacy and hinder the development of their emotional management skills (Liu et al., 2025). Emotional self-efficacy is essentially the combination of emotional regulation and self-efficacy. Self-efficacy is another effective intervention for Internet addiction, similar to how it regulates emotions. Therefore, emotional self-efficacy should also positively impact Internet addiction.

The influence of physical activity enjoyment on emotional self-efficacy is mainly reflected in improving individuals’ confidence in emotion management and improving their ability to regulate emotions. Previous studies have shown that there is a significant positive correlation between physical activity and self-efficacy (Wang et al., 2020). For example, a study of adolescent drug abuse treatment found that adolescents who enjoyed physical activity performed better in terms of self-esteem, physical health, and effectiveness in preventing relapse. This finding suggests that the physical activity enjoyment is closely related to emotional self-efficacy, and that physical activity enjoyment help improve an individual’s self-efficacy and mental health (Furzer et al., 2021). In addition, there is study that indicate that physical activity enjoyment can not only significantly improve an individual’s emotional regulation ability, but also further enhance their emotional self-efficacy (Tang et al., 2022). Based on the above conclusions, this study suggests that adolescents’ enjoyment of sports can affect their own emotional self-efficacy, which in turn affects Internet addiction, so hypothesis 2 is proposed.

*H2*: Soccer physical activity enjoyment and IA are mediated by emotional self-efficacy among adolescents.

### 1.3 Mediating role of athlete participation in soccer

The enjoyment of sports in the early years of adolescence is of irreplaceable importance to enhance their participation in school sports activities (Gray et al., 2019). This is because physical activity enjoyment is an important prerequisite for athlete participation, when adolescents have a love and enjoyment of soccer, they will be more active and more active in soccer. As a result, there is a favorable correlation between athletes’ participation and their enjoyment of physical exercise. Enjoyment of physical activity is one of the key elements that encourages adolescents to do sports (Cairney et al., 2012; Atkins et al., 2015). When adolescents experience enjoyment from sports, they are more likely to continue to participate in and enjoy the joy of sports, and this positive experience can not only improve an individual’s physical health but also have a positive impact on their mental health (Liddle et al., 2017). Meanwhile, athlete participation can predict adolescents’ enjoyment of sports to a certain extent (Dunker et al., 2020).

Studies have shown a significant negative correlation between athlete participation and Internet addiction. This means that people who are more physically active tend to have lower symptoms of Internet addiction. Therefore, it has been proved that athlete participation can effectively alleviate the symptoms of Internet addiction (Chen et al., 2023). Some scholars have found from experimental results that exercise not only aids in the adaptive reshaping of reward circuits but also effectively reduces internet addiction among adolescents (Zhang et al., 2023). Specifically, exercise can activate the same reward circuits as internet addiction, allowing adolescents to experience similar feelings of pleasure and satisfaction, thereby reducing the positive reinforcement from addictive sources (Liu and Wang, 2020). Physical activities can reduce the release and utilization of dopamine in the body. High levels of dopamine are a key factor in psychological dependence and the recurrence of addiction (Wang et al., 2021). Additionally, since internet addiction can lead to a lack of self-control among adolescents, studies have shown that physical exercise helps improve their self-control (Yu and Mu, 2024). Based on previous research conclusions, this study believes that adolescents will athlete participate in soccer based on the physical activity enjoyment of soccer, so as to reduce the phenomenon of Internet addiction. Therefore, hypothesis 3 is proposed.

*H3*: Soccer physical activity enjoyment and IA are mediated by soccer athlete participation among adolescents.

### 1.4 Chain mediation role of soccer physical activity enjoyment and IA

**Figure 1:**
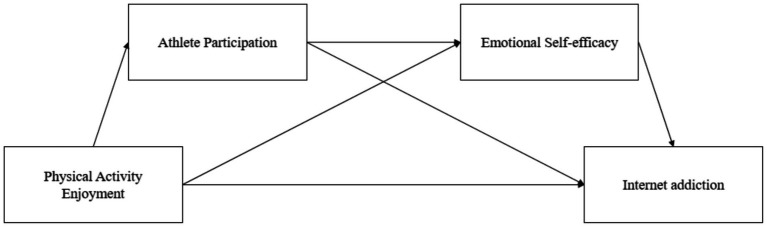
Hypothetical model diagram.

As one of the most popular sports in the world, adolescent soccer players can reduce their stress levels through positive relationships with their teammates. In relevant studies, the positive relationship between physical activity enjoyment and athlete participation has been revealed, and this relationship plays a key role in promoting youth athlete participation (Guan et al., 2023). In the process of participating in soccer, adolescents can constantly adjust the positive emotions of their teammates, and improve their emotional self-efficacy. This helps to minimize negative emotions among adolescents (Tamminen et al., 2016). Athlete participation represents a lasting, stable sports experience that encompasses a broad range of positive emotions and perceptions of an individual toward all sports (Zhang and Qian, 2022). Sports play a vital role in promoting students’ physical and mental health, but to achieve this goal, students’ active participation is an indispensable prerequisite. Meanwhile, the improvement of athlete participation helps to enhance the emotional self-efficacy of individuals. Emotional self-efficacy can be reformulated as a self-assessment or belief that adolescents hold about their ability to effectively manage negative emotions while addressing various challenges and to actively express emotions while enjoying enjoyable moments (Bandura et al., 2003; Cattelino et al., 2023). By participating in soccer, adolescents can learn how to face challenges overcome difficulties, and experience success and a sense of accomplishment in the process, thus improving their emotional well-being. In the process of participating in soccer, adolescents can vent their negative emotions and then better control their emotions (Pavlovıc et al., 2024). Researchers studied 505 Palestinian students to explore the relationship between Internet use and self-efficacy (Berte et al., 2021). The findings indicate a significant negative correlation between self-efficacy and Internet addiction patterns. Other scholars have found that adolescents with Internet addiction often struggle with emotional regulation, leading to a conclusion that there is a negative correlation between emotional regulation and Internet addiction (Giordano et al., 2023). Based on the above conclusions, this study believes that teenagers can better participate in football activities according to their preferences for football. In the process of participating in sports, they will have an impact on their own emotional self-efficacy and enhance their ability to regulate negative emotions, thus affecting Internet addiction. Therefore, hypothesis 4 is proposed. As a result, the research hypothesis model was constructed as shown in Figure 1.

*H4*: Soccer physical activity enjoyment and IA are mediated by soccer athlete participation and emotional self-efficacy among adolescents.

### 1.5 The present study

Although some studies have addressed the effects of sports on adolescent IA and the ability of team sports to affect adolescent IA (Sarı and Karagün, 2020; Zhang et al., 2023), how the soccer directly affects IA and its potential mediators is lacking. This study will focus on youth campus soccer participation and emotional self-efficacy to explore their relationship with IA. We expected to demonstrate the positive impact of soccer on IA and to reveal a mediating role of participation and emotional self-efficacy. The study will analyze how the enjoyment of soccer affects emotional self-efficacy, which will promote the overall development of adolescents and may reduce the phenomenon of IA.

## 2 Method

### 2.1 Participants

A total of 579 participants from 18 schools across Shaanxi Province, China were selected. They all participated in after-school training every day with the school soccer team. Each participant who knew the person took no more than 30 min to fill out the questionnaire, which was filled out from December to January 2023. After screening, 570 valid questionnaires were obtained (Table 1), resulting in a 98.45% validity rate. All participants signed a formal consent before filling out the questionnaires. Additionally, informed consent was also obtained from the parents of adolescents younger than 16 years. The methods for gathering data respected participants’ rights and followed ethical guidelines. This study was approved by the Ethics Committee of Shaanxi Normal University (NO. 202416014).

**Table 1:** Demographic information.

Variables	Numbers	Percentages	Variables	*M* ± SD
Gender				
Male	310	54%	Weekly training hours (by hours)	
Female	260	46%	Primary	3.942 ± 3.189
Period of study (age)				
Primary (8–12)	411	72%	Junior high	3.421 ± 1.486
Junior high (12–15)	159	28%	Training years (by years)	
Place of residence			Primary	2.082 ± 1.216
City	365	64%		
Village	205	36%	Junior high	4.283 ± 3.389

### 2.2 Procedure

With permission from each school, the questionnaires were given to classes of these adolescents. Our research team gave the participants a thorough explanation of the goal, importance, and questionnaire guidelines before the study’s commencement. This was done to make sure that the participants were aware that their comments would remain anonymous.

### 2.3 Measures

#### 2.3.1 Physical activity enjoyment scale (PACMS)

The PACMS was developed using Kendzierski and DeCarlo (1991). In China, the version translated by Sun (2014) is suitable for China’s national conditions, and it has good reliability and validity in other related studies. In this study, we adjusted the original content of the scale to translate those parts involving sports into more specific and focused soccer-related content. The scale is a single-dimensional scale consisting of 16 questions, such as “Playing soccer gives me a strong sense of achievement,” and is scored using the Likert 5-point scale. Items 1, 4, 6, 8, 9, 10, 11, 14 and 15 of the scale were set to reverse scoring. The higher the score, the greater the subjects’ enjoyment in sports, ranging from “very inconsistent” to “very consistent,” with a score of 1–5.

#### 2.3.2 Athlete participation scale (APS)

The APS, developed by Agbuga et al. (2016). The entire scale consists of 13 questions, covering three dimensions: behavioral engagement (4 items, e.g., “I will try my best to study in soccer training classes”), emotional engagement (4 items, e.g., “I feel happy during soccer training classes”), and cognitive engagement (5 items, e.g., “I will try to get feedback from the PE teacher to determine if my behavior is correct during soccer training classes”). The scale was translated into Chinese by Zhang (2023). Each question is scored by the Likert5 level method, the higher the score, the more exercise participants participated, from “completely inconsistent” to “completely consistent” score of 1 to 5. The Cronbach *α* of the three dimensions were 0.856, 0.812 and 0.806.

#### 2.3.3 Regulatory emotional self-efficacy scale (RES)

The Regulatory Emotion Self-Efficacy Questionnaire was developed by Caprara et al. (2008) and revised by Wen et al. (2009). The questionnaire consists of 12 questions, covering three dimensions: positive emotions (four items, e.g., ‘When something pleasant happens, I express my happiness’), regulating depression (five items, e.g., ‘When I feel lonely, I can help myself avoid feeling depressed’), and regulating anger (three items, e.g., ‘When others deliberately provoke me, I can avoid getting angry’). Responses are given on a 5-point Likert scale, ranging from 1 = strongly disagree to 5 = strongly agree. Higher scores indicate better regulatory emotion self-efficacy. The Cronbach α of the three dimensions were 0.802, 0.727, and 0.721.

#### 2.3.4 Internet addiction diagnostic questionnaire (DQ)

The DQ was developed by Young (1998). The questionnaire comprises eight core items, each precisely targeting key aspects of internet usage behavior. It aims to comprehensively and deeply assess whether individuals exhibit tendencies or symptoms of internet addiction. These items not only cover the frequency and duration of internet use but also delve into how internet activities impact daily life, work, study, interpersonal relationships, and mental health, providing a solid foundation for accurately identifying internet addiction. The questionnaire includes 20 questions (e.g., “You will crave the next time you go online after one visit”). To more accurately diagnose the level of internet addiction among adolescent participants, this study has refined the scoring criteria. Each question uses the Likert 6-point scale (1 = strongly disagree, 2 = disagree, 3 = neutral, 4 = agree, 5 = strongly agree, 6 = strongly agree) to evaluate respondents’ perceptions of the issues.

### 2.4 Data processing

Statistical analysis was performed as follows: SPSS27.0 was used for preliminary entry and analysis of the original data, reliability and validity test of the questionnaire, and descriptive statistics and correlation analysis of the data were carried out. At the same time, a chain mediation model was constructed using model 6 in PROCESS program developed by Hayes (2012) was used, and the significance of the mediation effect was tested by the bias-corrected percentile Bootstrap method. Additionally, the theoretical hypothesis model was tested by estimating 95% confidence intervals for the mediating and moderating effects by a sample of 1,000 instances. Besides, the confirmatory factor analysis was validated using the Amos 28.0. At the same time, the covariates were not included in the regression model.

## 3 Results

Since the data in this study were collected by questionnaires, exploratory factor analysis was used to test possible common methodological biases. Using Harman’s single-factor test method, a total of 61 factors were tested, among which 11 factors were greater than 1. The first common factor explanation rate is 19.945%, which is less than 40%. Therefore, there is no significant common methodological bias in the data in this study.

This research tested all constructs for validity using confirmatory factor analysis (Nunnally, 1978). Meanwhile, the constructs were assessed for the reliability of the data using Cronbach’s *α* (Cronbach, 1951). All scales had good reliability and validity (Table 2).

**Table 2:** Reliability and validity of scales (*n* = 570).

Scales	Cronbach *α*	*χ*^2^/df	RMSEA	AGFI	GFI	TLI
PACMS	0.851	4.338	0.077	0.867	0.904	0.925
APS	0.915	4.227	0.075	0.900	0.932	0.930
RES	0.844	3.687	0.069	0.920	0.951	0.909
DQ	0.901	3.053	0.060	0.890	0.913	0.904

### 3.1 Descriptive statistics and correlation analysis among variables

Table 3 presents the descriptive statistics of the variables. The correlation analyses revealed significant negative correlations between physical activity enjoyment, athlete participation, emotional self-efficacy, and IA in adolescents (all *ps* < 0.01). There was a significant positive relationship between physical activity enjoyment, athlete participation, and emotional self-efficacy (all *ps* < 0.01).

**Table 3:** Descriptive statistical analysis.

Variables	M	SD	1	2	3	4
Physical activity enjoyment	4.435	0.482	1			
Athlete participation	4.138	0.670	0.472***	1		
Emotional self-efficacy	3.603	0.666	0.324***	0.362***	1	
Internet addiction	2.456	0.887	−0.250***	−0.311***	−0.295***	1

The mean (M) and standard deviation (SD) are calculated by dividing the total score by the individual item of scale. ***p < 0.001.

### 3.2 Intermediate effect test

The results of the regression analysis in Table 4 showed that physical activity enjoyment had a direct positive predictive effect on athlete participation (*β* = 0.472, *t* = 12.755, *p* < 0.001) and emotional self-efficacy (*β* = 0.197, *t* = 4.517, *p* < 0.001). Athlete participation had a direct positive predictive effect on emotional self-efficacy (*β* = 0.269, *t* = 6.163, *p* < 0.001). Physical activity enjoyment had no significant direct predictive effect on IA (*β* = −0.095, *t* = −2.110, *p* = 0.035). Athlete participation had a significant negative effect on IA (*β* = −0.196, *t* = −4.290, *p* < 0.001), and emotional self-efficacy had a significant negative effect on IA (*β* = −0.193, *t* = −4.555, *p* < 0.001).

**Table 4:** The regression analysis between physical activity enjoyment, athlete participation, emotional self-efficacy, and IA (*n* = 570).

Dependent variables	Independent variables	*R* ^2^	*F*	*β*	*t*
Athlete participation	Physical activity enjoyment	0.221	162.678	0.472	12.755***
Emotional self-efficacy	Physical activity enjoyment	0.158	54.447	0.197	4.517***
	Athlete participation			0.269	6.163***
Internet addiction	Physical activity enjoyment	0.137	31.137	−0.095	−2.110*
	Athlete participation			−0.196	−4.290***
	Emotional self-efficacy			−0.194	−4.555***

*p < 0.05, ***p < 0.001.

**Figure 2:**
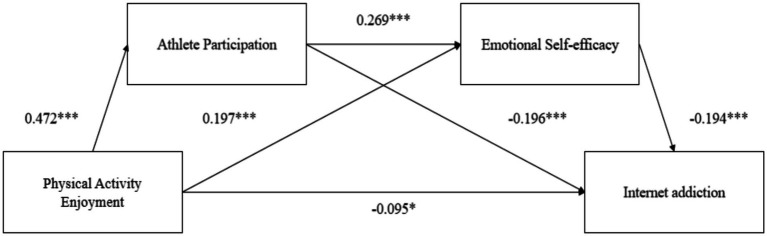
Chain mediating effect model of athlete participation and emotional self-efficacy between physical activity enjoyment and Internet addiction. *p < 0.05, **p < 0.01, ***p < 0.001.

As shown in Table 5, the results showed that athlete participation and emotional self-efficacy had a significant mediating effect, and the mediating effect was generated through three intermediary chains: First, soccer physical activity enjoyment → IA (Bootstrap 95%CI, [−0.421, −0.015]). Second, the indirect path of physical activity enjoyment → athlete participation → IA (Bootstrap 95%CI, [−0.378, −0.095]) suggested athlete participation significant mediated physical activity enjoyment and IA. Thirdly, for the indirect effect composed of physical activity enjoyment → emotional self-efficacy → IA (Bootstrap 95%CI, [−0.167, −0.022]) suggested emotional self-efficacy significant mediated physical activity enjoyment and IA. Fourth, the confidence interval of the indirect effect 3 composed of physical activity enjoyment → athlete participation → emotional self-efficacy → IA (Bootstrap 95%CI, [−0.107,-0.023]) suggested athlete participation and emotional self-efficacy significant mediated physical activity enjoyment and IA. Therefore, a theoretical model graph was constructed from the conclusions drawn from this study (Figure 2).

**Table 5:** Pathway analysis.

Paths	Effect value	SE	Boot-strap 95% CI	
Total effect	−0.575	0.093	−0.758	−0.391
Physical activity enjoyment → IA	−0.218	0.103	−0.421	−0.015
Physical activity enjoyment → Athlete participation → IA	−0.212	0.072	−0.378	−0.095
Physical activity enjoyment → Emotional self-efficacy → IA	−0.088	0.037	−0.167	−0.022
Physical activity enjoyment → Athlete participation → Emotional self-efficacy → IA	−0.057	0.021	−0.107	−0.023

Boot standard error, Boot CI lower limit, and Boot CI upper limit refer to the standard error, lower and upper limits of the percentile Boots-trap method, respectively. Three decimal places were retained for all digits.

## 4 Discussion

This study built a chain mediation model to thoroughly examine the link between physical activity enjoyment, athlete participation, emotional self-efficacy, and IA in adolescent soccer, drawing on prior research on sports and IA. It is found that physical activity enjoyment not only has a positive effect on IA through the chain mediating effect of athlete participation and emotional self-efficacy but also as independent mediating factors, athlete participation, and emotional self-efficacy can affect IA. This research has a significant impact on the theory and practice of preventing adolescent IA and encouraging the physical and mental development of adolescents.

### 4.1 Chain mediation role of soccer physical activity enjoyment and IA between physical activity and IA

This study found that soccer athlete participation and emotional self-efficacy play an independent mediating role in the relationship between soccer physical activity enjoyment and IA behavior, and at the same time, they influence adolescent IA behavior together as a chain intermediary, the H4 is supported.

Many studies have shown that physical exercise can have a negative correlation with IA behavior through some mediating effects (Liu et al., 2025; Qiu et al., 2023). In soccer sports participation, adolescents may gradually develop their interest in soccer and then continue to participate in soccer. Mutual encouragement and support among teammates in soccer participation can also enhance adolescents’ confidence and ability to deal with negative emotions and reduce the phenomenon of IA (McEwan and Beauchamp, 2014). From a positive perspective, it is generally believed that adolescents’ mental health can be well supported and their mental health literacy can be improved by participating in organized sports, especially team sports (Fromel et al., 2022). Especially in the early stage of youth, participating in soccer activities can bring adolescents positive emotional experiences and strong motivation for athlete participation, which will greatly motivate them to continue to participate in soccer in the future and improve the physical activity enjoyment of soccer (Zheng et al., 2023).

The study further demonstrated the internal mechanism of the influence of physical activity enjoyment on adolescents’ IA behavior, that is, adolescents constantly improve their interest in soccer in daily soccer and recognize the enjoyment brought by soccer as a team sport. This increase in positive emotions can help adolescents better distract their attention from the Internet. The negative emotions from the body can be reduced, and then emotional self-efficacy can be improved (Wienke and Jekauc, 2016). Continuous soccer athlete participation and the improvement of emotional self-efficacy jointly affect the generation of IA. Adolescents’ participation in soccer can enhance their self-regulation and self-control abilities (Yang et al., 2019), which are part of self-efficacy. It can be proved that adolescents’ athlete participation in soccer can improve their emotional self-efficacy and have a positive impact on IA. Simultaneously, numerous studies have clearly pointed out that physical activity plays a vital role in promoting mental health. Specifically, by athletes participating in soccer, adolescents can alleviate anxiety depression, and other negative emotions, and improve their emotional state effectively. It also improves the emotional self-efficacy of adolescents and fosters a healthy psychological state. The present study showed how beneficial soccer is for both physical and mental health and provided a useful strategy for enhancing mental health (Williams, 2008; Wipfli et al., 2008; Carek et al., 2011).

### 4.2 Mediating effect of emotional self-efficacy between physical activity and IA

This study found that emotional self-efficacy played a partial mediating role between soccer physical activity enjoyment and adolescent IA, which confirmed the establishment of H2. Adolescents using the Internet excessively may be a coping mechanism to get through stressful situations by socially isolating themselves, avoiding or seeking instant emotional pleasure, and diverting their attention (Kun and Demetrovics, 2010; Schreiber et al., 2012).

The more incapacitated adolescents are, the more likely they are to have Internet-addictive behaviors. The results of this study are consistent with Aydın (2017). As Spada et al. (2008) found, excessive use of the Internet may be a poor form of self-regulation that helps adolescents temporarily distract and relieve their negative emotional states. Soccer training can better help adolescents to relieve many negative emotions such as anxiety and pressure (Park et al., 2020). Their feeling of self-efficacy gives them the confidence to handle emotional difficulties in daily life in addition to helping them do well on the court. This also confirms from another aspect that improving emotional self-efficacy can play a positive role in IA.

### 4.3 Mediating role of athlete participation in soccer between physical activity and IA

Additionally, this study discovered that participation in soccer by athletes served to mediate the enjoyment of soccer physical activity and IA, supporting the validity of our study’s hypothesis H3 and corroborating the findings of previous studies that regular sports such as soccer sport can affect adolescents’ IA behavior (Lin et al., 2008; Koçak, 2019). Meanwhile, studies have also proved that there is a negative correlation between physical activity and IA, which is consistent with soccer athlete participation, further highlighting the potential benefits of physical activity in alleviating IA (Li Z. et al., 2023).

Relevant studies have revealed that spending a long time online can lead to neurological disorders in adolescents, which can lead to serious problems of IA. This disorder can cause adolescents to become addicted to the Internet, which can lead to IA. This study emphasizes how adolescents’ physical and mental health may be negatively impacted by excessive Internet usage, and how urgent it is to prevent and treat IA (Koepp et al., 1998). Studies have shown that a certain intensity of exercise promotes the brain and body’s physiological system through multiple stimuli, which can not only enhance the physical health of adolescents but also improve the nervous system function and relieve the symptoms of adolescents’ mobile phone addiction (Gormley et al., 2008). Soccer is also a team sport with a certain intensity. Combined with these two studies, it can be concluded that moderate participation in physical exercise such as soccer can not only allow adolescents to enjoy the physical activity enjoyment, improve self-control, and reduce Internet use time but also help improve nervous system function and prevent the occurrence of IA. These studies can prove that soccer athlete participation can have a good influence on IA behavior.

### 4.4 The effect of physical activity enjoyment in soccer on IA

The results show that there is a direct predictive effect between physical activity enjoyment and IA, supported H1. Cai et al. (2023) found that among male adolescents, sports activities and their interests show significant negative differences in online game addiction, which is consistent with what we have verified in our study. Studies have also found that soccer is more able to stimulate adolescents’ physical activity enjoyment than sprint because adolescents have more motivation to participate in sports when they participate in soccer than in sprint (Hammami et al., 2023). It is also mentioned in relevant studies that team sports can reduce the occurrence of IA behavior. Meanwhile, schools and families should cultivate students’ interest in soccer, to reduce IA behavior (Zhang et al., 2023). Therefore, to reduce the IA phenomenon, adolescents can be more actively involved in soccer activities by enhancing their interest in soccer.

The IA is a kind of unhealthy mental behavior, and physical activity enjoyment plays a key role in improving adolescent mental health, thus helping to reduce the risk of IA (Sarı and Karagün, 2020). When youth physical activity enjoyment from soccer, they receive positive emotional feedback, and their self-confidence and self-efficacy increase (Donaldson and Ronan, 2006). These positive changes in mental health will develop the courage and determination of adolescents to face challenges and difficulties in real life, reduce excessive dependence on the virtual world of the network, and thus reduce the possibility of IA. Studies have also proved that physical activity enjoyment has a direct positive impact on IA (Doré et al., 2022). By promoting the enjoyment of soccer, we can effectively prevent and reduce the occurrence of IA, which is consistent with the results of this study.

## 5 Limitations and future studies

First, due to the cross-sectional design, it is challenging to establish causal relationships between variables. Future studies could adopt experimental methods or longitudinal tracking designs to more accurately reveal the causal pathways and mediating mechanisms among variables. Second, the sample spans from primary to secondary school, with a wide age range that may affect the specificity of the results. Future research could focus on specific age groups (such as early adolescence) to enhance the applicability of intervention measures. Finally, it is recommended to incorporate socioeconomic background variables (such as parents’ education levels and family income) and other types of extracurricular activities (such as arts and science) into the analysis of moderating effects, to build a more comprehensive theoretical model. These improvements will deepen our understanding of how the physical enjoyment of soccer influences the mechanism of adolescent internet addiction and provide empirical evidence for the development of differentiated intervention strategies.

## 6 Conclusion

This study has constructed a chain mediation model, which explores the mediating role of athlete participation and emotional self-efficacy between physical activity enjoyment and IA. The findings suggest that greater physical activity enjoyment can directly mitigate IA, and it can also alleviate the phenomenon of IA among adolescents through both athlete participation and emotional self-efficacy. Moreover, physical activity enjoyment can also alleviate IA separately for adolescents through either a higher level of athlete participation or emotional self-efficacy.

## Data Availability

The datasets presented in this study can be found in online repositories. The names of the repository/repositories and accession number(s) can be found at: 10.6084/m9.figshare.28103534, https://figshare.com/articles/dataset/_570_sav/28103534?file=51398171.
